# Excited-state fidelity as a signal for the many-body localization transition in a disordered Ising chain

**DOI:** 10.1038/s41598-017-00660-4

**Published:** 2017-04-03

**Authors:** Taotao Hu, Kang Xue, Xiaodan Li, Yan Zhang, Hang Ren

**Affiliations:** 1 School of Physics, Northeast Normal University, Changchun, 130024 People’s Republic of China; 2 College of Science, University of Shanghai for Science and Technology, Shanghai, 200093 People’s Republic of China; 3 Key Laboratory of Airborne Optical Imaging and Measurement, Changchun Institute of Optics, Fine Mechanics and Physics, Chinese Academy of Sciences, Changchun, 130033 People’s Republic of China

## Abstract

In this work, we use exact matrix diagonalization to explore the many-body localization (MBL) transitions in quantum Ising chains with disordered nearest-neighbour couplings, disordered next-nearest-neighbour couplings and disordered external fields. It is demonstrated that the fidelity can be used to characterize the interaction-driven MBL transition in this closed spin system in a manner that is consistent with previous analytical and numerical results. We compute the fidelity for high-energy many-body eigenstates, namely, the excited-state fidelity. It is demonstrated that disordered nearest-neighbour couplings, disordered next-nearest-neighbour couplings and disordered external fields each have different effects on the MBL transition. Furthermore, we investigate the MBL transition of a quantum Ising chain with both disordered nearest-neighbour couplings and disordered next-nearest-neighbour couplings to see how these two types of disordered couplings drive the occurrence of the MBL transition.

## Introduction

The concept of Anderson localization has been well established since Anderson proposed it in his seminal paper^1^ more than half a century ago. This concept states that a static disordered potential can lead to a complete absence of diffusion in a closed quantum system. This idea has received considerable attention since its proposal and has ultimately led to the conclusion that non-interacting systems in one and two dimensions will be localized for arbitrary disorder, even when that disorder is very small^2^. In ref. 1, Anderson also conjectured that a closed interacting quantum system with sufficiently strong disorder would fail to approach thermal equilibrium. Much more recently, Basko *et al*.^3^ presented new arguments to revive this idea of many-body localization (MBL). MBL is a quantum “glass transition” that occurs at nonzero (or even infinite) temperature, where equilibrium quantum statistical mechanics breaks down. In the localized phase, the system fails to thermally equilibrate. Like the more familiar ground-state quantum-phase transitions, this transition is a sharp change in the properties of the many-body eigenstates of the Hamiltonian; unlike ground-state phase transitions, the MBL transition at nonzero temperature appears to be only a dynamical phase transition that is invisible in equilibrium thermodynamics^4^.

Many recent studies^4–17^ have investigated and confirmed the phenomenon of MBL, showing that a novel dynamical phase transition can occur in interacting disordered systems. Many features of the many-body localized phase have been explored. It has been demonstrated that the bipartite entanglement entropy between two sectors of such a system shows a characteristic logarithmic growth in the many-body localized phase^18–23^. It has also been found that the total correlation scales strongly with the localized phase, exhibiting a pronounced peak at the transition^15^.

Disorder is an intrinsic property of all real systems. The interplay between disorder and interaction is a leitmotif of condensed matter physics; it constitutes the driving mechanism of the “glass transition” (metal-insulator transition). Therefore, in this work, we investigate the MBL transition in a quantum Ising chain with disordered couplings to explore how the interplay between disorder and interaction drives the MBL transition. In particular, we investigate the effects of not only disordered nearest-neighbour couplings but also disordered next-nearest-neighbour couplings, which here introduce a repulsive interaction between domain walls on adjacent bonds in the quantum Ising chain.

Meanwhile, considerable efforts^24–37^ have recently been devoted to investigating the role of a popular concept in quantum information theory, namely, fidelity in quantum critical phenomena^38^. It has been demonstrated that fidelity is useful for characterizing distinct phases of quantum many-body systems^39^. In particular, the minimum in the fidelity near a critical point has been studied in several models^24, 25^. It has also been shown that fidelity plays a crucial role in quantum phase transitions (QPTs) in quantum fields^40^. In particular, both fidelity and the Berry phase have recently been used to analyse QPTs from a geometric perspective. In ref. 33, Venuti *et al*. unified these two approaches, showing that the underlying mechanism is the critical singular behaviour of a complex tensor over the Hamiltonian parameter space. The advantage of the fidelity is that it is a spatial geometric quantity; no a priori knowledge of the order parameter or symmetry breaking is required in studies of QPTs.

Considering the unique and crucial role of fidelity in quantum critical phenomena, in this work, we apply the fidelity approach to the study of MBL transitions. In ref. 26 the authors applied the fidelity approach to estimate random transitions of a disordered quantum model. They showed that the fidelity susceptibility and its scaling properties provide useful information about the phase diagram. Thus, the purpose here is phenomenological; we believe that this approach should work based on previous analysis^24, 26^, and we do the numerics and see that it does indeed work. Since MBL involves all energies and, recently, has been widely studied for high-energy states^4–9^, the states in the middle of the spectrum are important. Therefore, here, instead of the ground state, which represents a low temperature, we consider only high-energy states; namely, our focus is on the excited-state fidelity. Thus, fingerprints of the MBL transition are expected to be observed in the fidelity of the excited states. Following ref. 24 the ground-state fidelity is defined as the overlap between $|{{\rm{\Psi }}}_{0}(\lambda )\rangle$ and $|{{\rm{\Psi }}}_{0}(\lambda +\delta \lambda )\rangle$, i.e.,

1 $${F}_{0}(\lambda ,\lambda +\delta \lambda )=\langle {{\rm{\Psi }}}_{0}(\lambda )|{{\rm{\Psi }}}_{0}(\lambda +\delta \lambda )\rangle .$$

Similarly, the fidelity of the n-th excited state $|{{\rm{\Psi }}}_{n}(\lambda )\rangle$ of the system is defined as the overlap of the excited states with parameters *λ* and *λ* + *δλ*:

2 $${F}_{n}(\lambda ,\lambda +\delta \lambda )=\langle {{\rm{\Psi }}}_{n}(\lambda )|{{\rm{\Psi }}}_{n}(\lambda +\delta \lambda )\rangle .$$

## Numerical model

The Hamiltonian of a one-dimensional quantum Ising chain with nearest-neighbour couplings and next-nearest-neighbour couplings in an external field in the z direction reads as follows:

3 $$H=-\sum _{i=1}^{N-1}{J}_{i}{\sigma }_{i}^{z}{\sigma }_{i+1}^{z}-\sum _{i=1}^{N-1}{K}_{i}{\sigma }_{i}^{z}{\sigma }_{i+2}^{z}+{h}_{i}{\sigma }_{i}^{z},$$

where ${\sigma }_{i}^{z}$ is the Pauli matrix acting on the *i*-th qubit; *N* denotes the total number of sites; *J*_*i*_ and *K*_*i*_ are the nearest-neighbour coupling and next-nearest-neighbour coupling, respectively, at site *i*; and *h*_*i*_ represents the static external field at site *i*. In the present work, we are interested in the regime of repulsive interactions in a quantum Ising chain. We will study the features of the excited-state fidelity for the model given in (3) in four cases: (a) The *J*_*i*_ are independent random variables at each site *i*, each with a probability distribution that is uniform in [−*J*, *J*], whereas *K*_*i*_ and *h*_*i*_ are constant, −*K*_*i*_ = *h*_*i*_ = 0.5. (b) The *K*_*i*_ are independent random variables at each site *i*, each with a probability distribution that is uniform in [−*K*, *K*], whereas *J*_*i*_ and *h*_*i*_ are constant, −*J*_*i*_ = 2*h*_*i*_ = 1. (c) The *h*_*i*_ are independent random variables at each site *i*, each with a probability distribution that is uniform in [−*h*, *h*], whereas *K*_*i*_ and *J*_*i*_ are constant, *J*_*i*_ = −2*K*_*i*_ = 1. (d) Both the *J*_*i*_ and the *K*_*i*_ are independent random variables at each site *i*, each with a probability distribution that is uniform in [−*J*, *J*] or [−*K*, *K*], respectively, whereas *h*_*i*_ is constant, *h*_*i*_ = 0.5. Thus, we compute the excited-state fidelities *F*_*n*_(*J*, *J* + *δJ*), *F*_*n*_(*K*, *K* + *δK*), *F*_*n*_(*h*, *h* + *δh*) and *F*_*n*_(*J*, *K*; *J* + *δJ*, *K* + *δK*), respectively, for these four cases. Notably, for disordered *J*_*i*_, disordered *K*_*i*_ and disordered *h*_*i*_, the parameter perturbations *δJ*_*i*_, *δK*_*i*_ and *δh*_*i*_ at each site that are used in this paper are not determinate; instead, they, like *J*_*i*_, *K*_*i*_ and *h*_*i*_, are random variables drawn from uniform random distributions in [−*J*, *J*], [−*K*, *K*] and [−*h*, *h*], respectively, with the following forms: *δJ*_*i*_ = *εJ*_*i*_, *δK*_*i*_ = *εK*_*i*_ and *δh*_*i*_ = *εh*_*i*_ (*ε* is a small constant). In cases (a), (b) and (c), let *ε* = 10^−5^; in case (d), let *ε* = 10^−3^. Then, for each disorder realization, we find the many-body eigenstates $|{{\rm{\Psi }}}_{n}\rangle$ that are in the middle third of the energy-ordered list of all data. Our qualitative conclusions do not depend on the exact values of these parameters. We then compute the fidelity *F*_*n*_ for each eigenstate $|{{\rm{\Psi }}}_{n}\rangle$. Averaging over all selected excited states and disorder realizations yields the mean value E[*F*]. The numerical analyses were performed using standard libraries for exact matrix diagonalization. For all parameter values, this model has two global conservation laws: one for the total energy and one for the total magnetization *S* ^*z*^ along the z direction. The total *S* ^*z*^ symmetry and parallel programming techniques were employed to make the computations feasible. For each disorder amplitude $|J|$, $|K|$ and $|h|$, we used 10^4^ disorder realizations for *N* = 6 and *N* = 8, 2000 realizations for *N* = 10 and *N* = 12, 200 realizations for *N* = 14, and 50 realizations for *N* = 16 to obtain the data shown in this paper.

**Figure 1:**
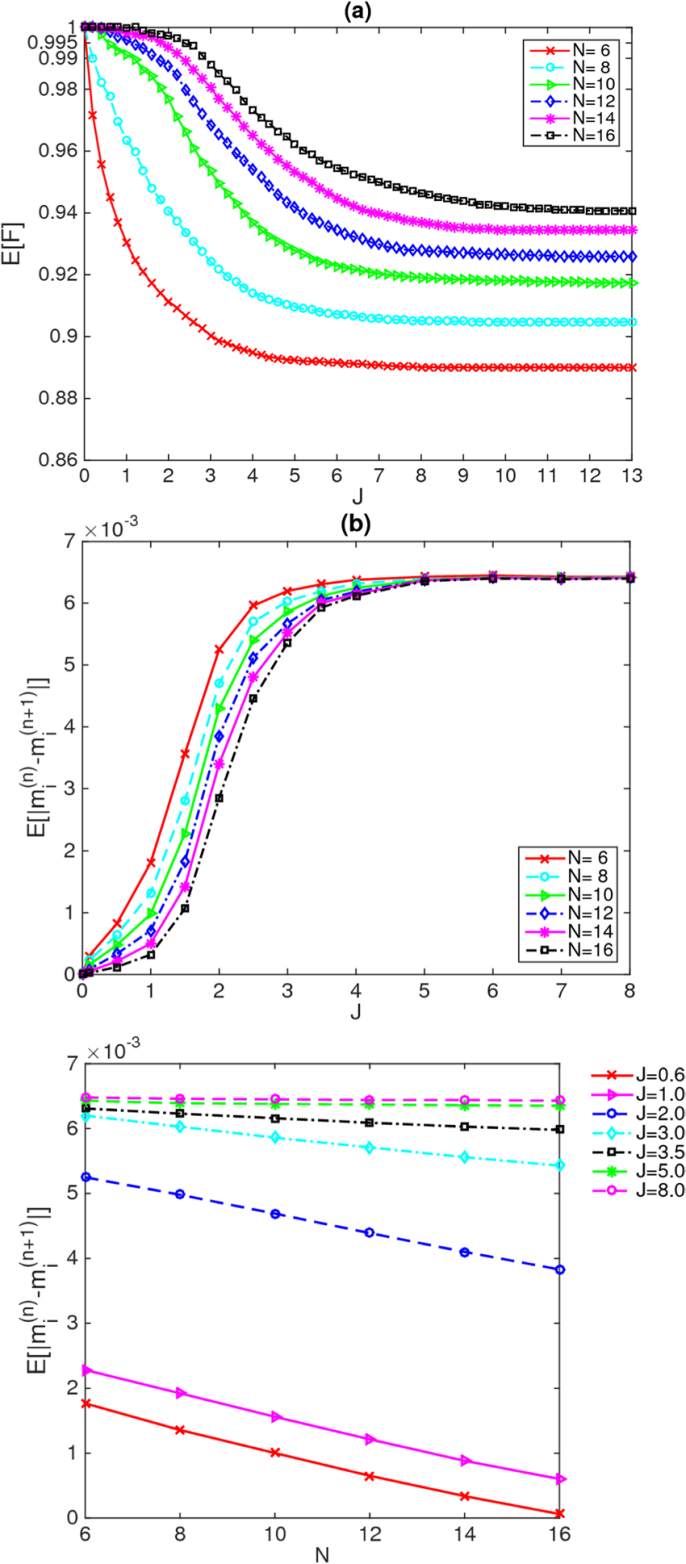
(**a**) Average fidelity as a function of the disorder strength *J* for system sizes from 6 to 16. The system sizes *N* are indicated in the legend. (**b**) Average difference as a function of the disorder strength *J*. (**c**) Average difference as a function of the system size *N* for different values of the disorder strength *J*. The values of *J* are indicated in the legend. In the ergodic phase (at small *J*), where the eigenstates are thermal, these differences vanish exponentially in *N* as *N* increases, whereas they remain large in the localized phase (at large *J*).

**Figure 2:**
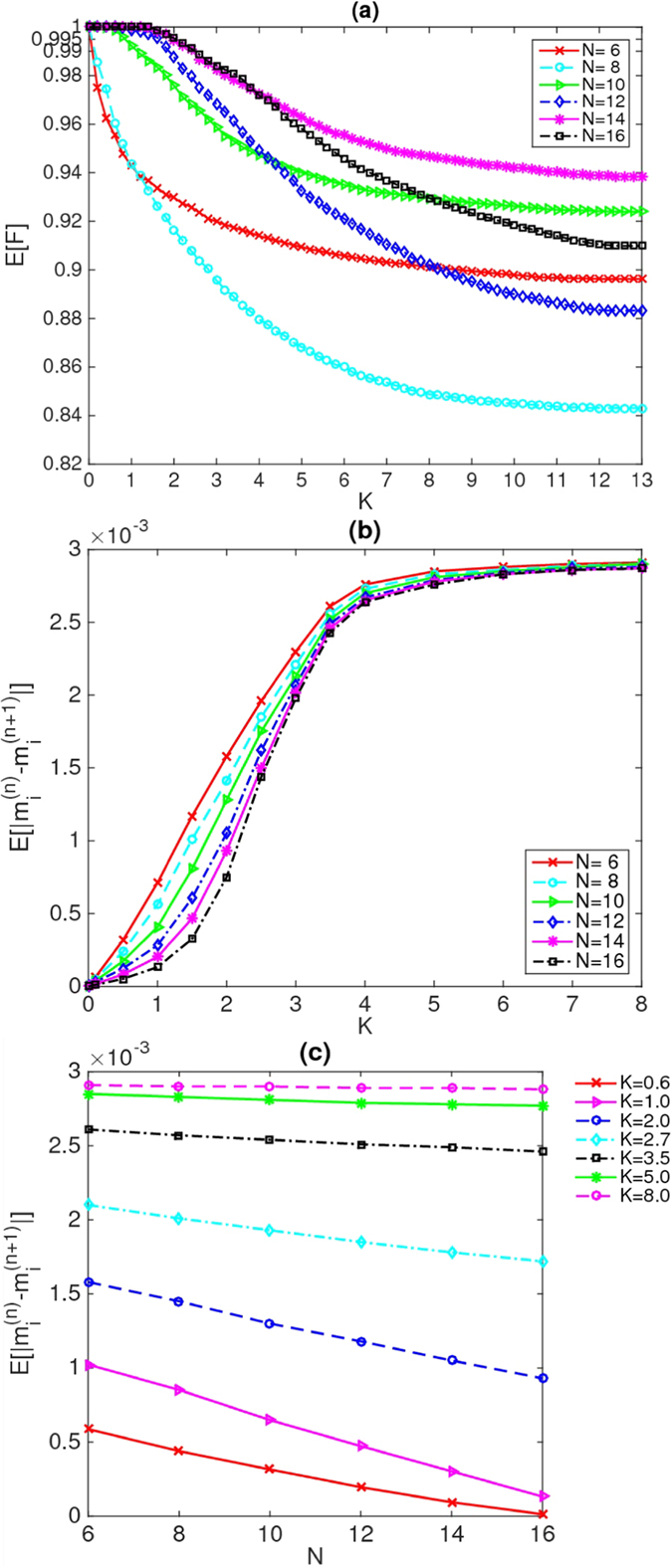
(**a**) Average fidelity as a function of the disorder strength *K* for system sizes from 6 to 16. The system sizes *N* are indicated in the legend. (**b**) Average difference as a function of the disorder strength *K*. (**c**) Average difference as a function of the system size *N* for different values of the disorder strength *K*. The values of *K* are indicated in the legend. In the ergodic phase (at small *K*), where the eigenstates are thermal, these differences vanish exponentially in *N* as *N* increases, whereas they remain large in the localized phase (at large *K*).

**Figure 3:**
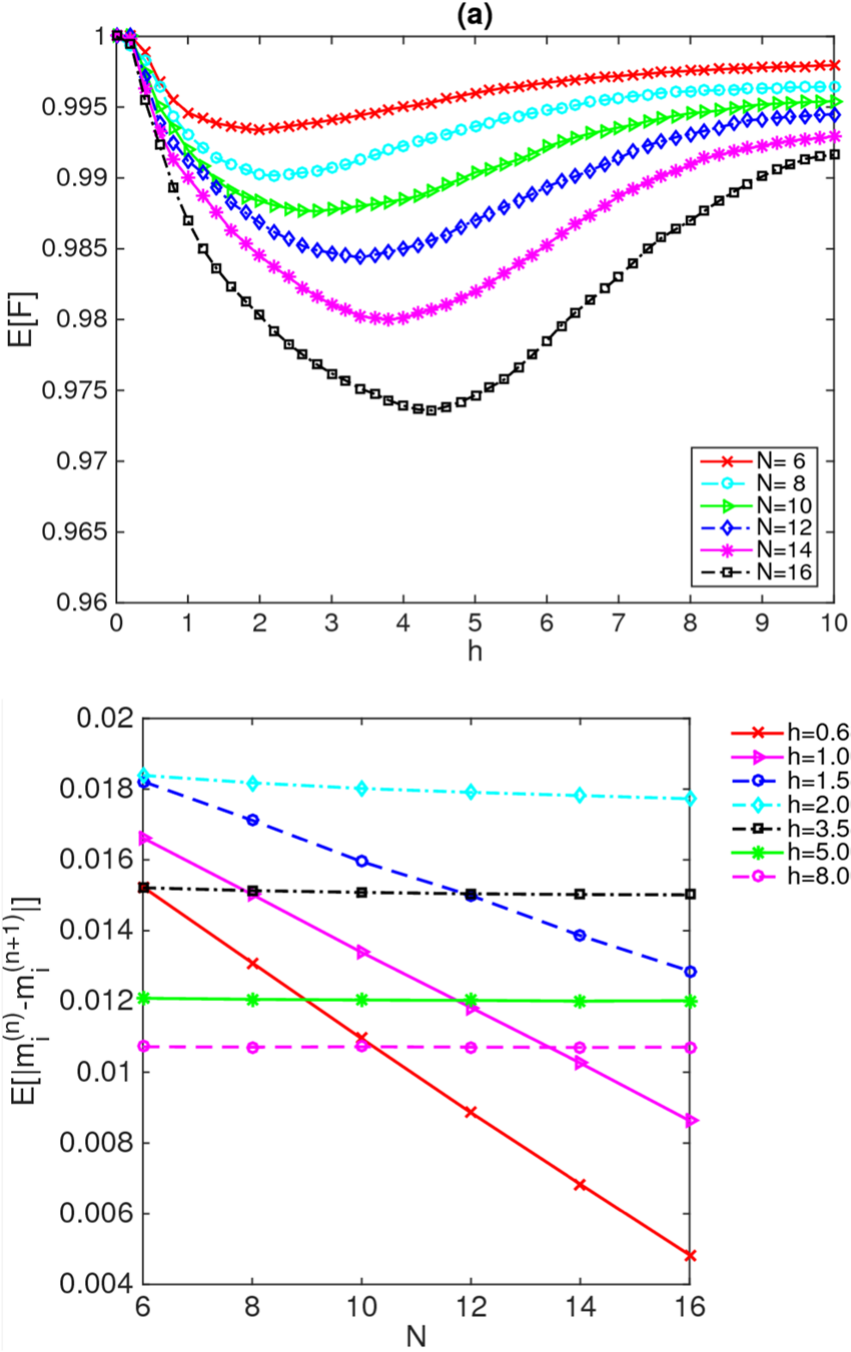
(**a**) Average fidelity as a function of the disorder strength *h* for system sizes from 6 to 16. The system sizes *N* are indicated in the legend. In the ergodic phase (at small *h*), E[*F*] decays substantially under the dynamics until *h* approaches the critical point; then, in the localized phase (at large *h*), E[*F*] increases again to approximately approach 1. (**b**) Average difference as a function of the system size *N* for different values of the disorder strength *h*. The values of *h* are indicated in the legend. In the ergodic phase (at small *h*), where the eigenstates are thermal, these differences vanish exponentially in *N* as *N* increases, whereas they remain large in the localized phase (at large *h*).

In Figs 1(a), 2(a) and 3(a), we plot the averaged excited-state fidelity E[*F*] as a function of each of the disorder strengths *J*, *K* and *h*, respectively, for the energies in the middle third of the spectrum. We can see pronounced differences among the data in these three figures. It is known that in the ergodic phase ($J < {J}_{c}$, $K > {K}_{c}$ and $h < {h}_{c}$), the many-body eigenstates are thermal^41–43^, and consequently, the isolated quantum system can relax to thermal equilibrium under the dynamics of its Hamiltonian. By contrast, in the many-body localized phase ($J > {J}_{c}$, $K > {K}_{c}$ and $h > {h}_{c}$), the many-body eigenstates are not thermal^3^; consequently, the “eigenstate-thermalization hypothesis”^41–43^ is false in the localized phase. Thus, in the localized phase, the isolated quantum system does not relax to thermal equilibrium under the dynamics of its Hamiltonian. To obtain further confirmation that the MBL transition really occurs and to identify the critical points *J*_*c*_, *K*_*c*_ and *h*_*c*_ for the model given in (3), we also compute, for each disorder realization, the corresponding local expectation value of the *z* component of the spin

4 $${m}_{i}^{(n)}=\langle {{\rm{\Psi }}}_{n}|{s}_{i}^{z}|{{\rm{\Psi }}}_{n}\rangle $$

at site *i* in the eigenstate $|{{\rm{\Psi }}}_{n}\rangle$. For each site in each disorder realization, we compare these expectation values for eigenstates that are adjacent in energy; in the first three cases, averaging over all selected excited states, disorder realizations, and sites yields the mean value of the difference $E[|{m}_{i}^{(n)}-{m}_{i}^{(n+\mathrm{1)}}|]$ for various *J*, *K* and *h*, respectively, where the eigenstates are indexed by *n* in the order of their energies. The selected many-body eigenstates are those in the middle third of the energy-ordered list of states. In this energy range, the difference $E[|{m}_{i}^{(n)}-{m}_{i}^{(n+\mathrm{1)}}|]$ in energy density between adjacent states $|{{\rm{\Psi }}}_{n}\rangle$ and $|{{\rm{\Psi }}}_{n+1}\rangle$ is of order $\sqrt{N}{2}^{-N}$ and thus becomes exponentially small in *N* as *N* increases. If these eigenstates are thermal, then they represent temperatures that differ only by this exponentially small amount; therefore, the expectation values of ${s}_{i}^{z}$ for two such states should be the same for $N\to \infty$.

In Figs 1(b) and 2(b), we plot the mean value of the difference $E[|{m}_{i}^{(n)}-{m}_{i}^{(n+\mathrm{1)}}|]$ as a function of each of the disorder strengths *J* and *K*, respectively, for energies in the middle third of the spectrum. In Figs 1(c), 2(c) and 3(b), we also plot the mean value of the difference $E[|{m}_{i}^{(n)}-{m}_{i}^{(n+\mathrm{1)}}|]$ as a function of the system size *N* for different values of the disorder strengths *J*, *K* and *h*, respectively.

For disordered couplings *J* and *K*, from Figs 1(c) and 2(c), one can see that the differences $E[|{m}_{i}^{(n)}-{m}_{i}^{(n+\mathrm{1)}}|]$ do indeed appear to be exponentially decreasing with increasing *N* in the ergodic phase (at small *J* and *K*, respectively), as expected. By contrast, in the localized phase (at large *J* and *K*, respectively), the differences $E[|{m}_{i}^{(n)}-{m}_{i}^{(n+\mathrm{1)}}|]$ between adjacent eigenstates remain large as *N* increases, confirming that these many-body eigenstates are not thermal, namely, that they are localized at large disorder strengths *J* and *K*. These findings indicate that the disordered couplings drive the occurrence of the MBL transition in this model. Accordingly, we can obtain *J*_*c*_ ∈ (3, 4) and *K*_*c*_ ∈ (2.7, 4), respectively, for the breakdown of the ergodic phase. In Figs 1(a) and 2(a), one can see that with increasing disorder, the excited-state fidelity decreases and eventually saturates at a nearly stable value, where the critical point depends on the system size. Meanwhile, in Figs 1(b) and 2(b), the difference $E[|{m}_{i}^{(n)}-{m}_{i}^{(n+\mathrm{1)}}|]$ increases and also eventually saturates at a nearly stable value with increasing disorder; again, the critical point is size dependent. By comparing Fig. 1(a) and (b) and also Fig. 2(a) and (b), one can see that the transition regions for the excited-state fidelity and the difference $E[|{m}_{i}^{(n)}-{m}_{i}^{(n+\mathrm{1)}}|]$ are consistent. Thus, the excited-state fidelity effectively signals the MBL transition in such a disordered Ising chain. Although both disordered nearest-neighbour couplings and disordered next-nearest-neighbour couplings can drive the occurrence of the MBL transition, their effects on the MBL transition somewhat differ. In Fig. 1(a), for disordered nearest-neighbour couplings *J*, the value of the critical point decreases and the drop becomes sharper as the system size *N* decreases. Interestingly, Fig. 2(a) shows that for disordered next-nearest-neighbour couplings *K*, two distinct behaviour regimes are evident: for system sizes of 6, 10 and 14 and for system sizes of 8, 12 and 16. Within each of these groups, however, the same behaviour is observed: the value of the critical point decreases and the drop becomes sharper as the system size *N* decreases. A comparison between Figs 1(a) and 2(a) reveals that for system sizes of 6, 10 and 14, the curves of the excited-state fidelity as a function of the disorder strengths *J* and *K* have similar shapes, whereas for system sizes of 8, 12 and 16, the curves for the two disorder strengths *J* and *K* are different: the drops are sharper for the next-nearest-neighbour couplings *K*. However, the reason for this interesting distinction is not yet clear. We will research this problem further in our future work.

Meanwhile, for disordered external fields, Fig. 3(b) indicates that the differences $E[|{m}_{i}^{(n)}-{m}_{i}^{(n+\mathrm{1)}}|]$ also appear to be exponentially decreasing with increasing *N* in the ergodic phase (at small *h*), as expected. Moreover, in the localized phase (at large *h*), the differences $E[|{m}_{i}^{(n)}-{m}_{i}^{(n+\mathrm{1)}}|]$ between adjacent eigenstates again remain large as *N* increases; as in the cases of the disordered couplings discussed above, this observation confirms that these many-body eigenstates are not thermal but rather are localized at large disorder strengths *h*. This finding indicates that disordered external fields can also drive the occurrence of the MBL transition. Accordingly, we obtain *h*_*c*_ ∈ (2, 3.5) for the breakdown of the ergodic phase, which is consistent with the predictions presented in refs 5 and 11 and in our recent work^17^. In Fig. 3(a), the curve of E[*F*] versus *h* shows, at low *h*, an initial increase towards a minimum; then, at higher *h*, it increases to approximately approach 1. In other words, the excited-state fidelity goes to 1 or remains unchanged when deep in either the thermal or the localized phase but diverges at the transition. This diverging valley might be understood as a consequence of the many-body mobility edge. The critical point again depends on the system size; the behaviour of the transition region for the excited-state fidelity is consistent with that of the difference $E[|{m}_{i}^{(n)}-{m}_{i}^{(n+\mathrm{1)}}|]$. This observation indicates that the excited-state fidelity can effectively signal the MBL transition in a disordered Ising chain of this type, as well.

**Figure 4:**
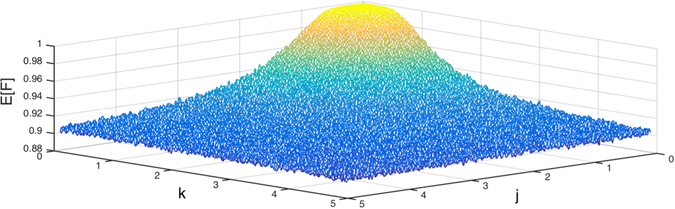
Average fidelity as a function of both *J* and *K* for a system of size 8. The number of disorder realizations for each disorder amplitude $|J|$ and $|K|$ is 10^4^. Under these dynamics, E[*F*] decays substantially within a certain range of the *(J*, *K*) parameter space and then gradually tends towards stability.

Finally, in Fig. 4, we plot the average excited-state fidelity as a function of both *J* and *K* for a system of size 8 to see how these two types of couplings drive the MBL transition to occur. The plot shows that under these dynamics, E[*F*] decays substantially within a certain range of the (*J*, *K*) parameter space and then gradually tends towards stability. The edge of this range of the (*J*, *K*) parameter space might also be understood as a consequence of the many-body mobility edge.

## Summary

In this paper, we use exact matrix diagonalization to explore the many-body localization (MBL) transitions in quantum Ising chains with disordered nearest-neighbour couplings, disordered next-nearest-neighbour couplings and disordered external fields. Numerical simulations show that the excited-state fidelity can be used to characterize the interaction-driven MBL transition in a unique way that is nevertheless consistent with previous analytical and numerical results^24, 26^. We test the fidelity between two excited states related by a small parameter perturbation *δJ*, *δK*, *δh* or (*δJ*, *δK*); notably, the parameter perturbation at each site that is considered here is also a random variable. Disorder is an intrinsic property of all real systems, and the interplay between disorder and interaction constitutes the driving mechanism of the glass transition (metal-insulator transition); similarly, the transition from the ergodic to the many-body localized phase is a highly non-equilibrium phenomenon, but one that is poorly understood at present. Our study of the exact matrix diagonalization of the model given in (3) allows us to partially explore how the interplay between disorder and interaction drives the occurrence of the MBL transition and elucidates some of the properties of the ergodic and localized phases through an examination of some of the properties of the many-body eigenstates of our finite-size systems in the vicinity of the localization transition. The results show that for this model with disordered couplings, the excited-state fidelity exhibits a pronounced drop at the transition and then gradually tends towards stability in the localized phase, with a critical point that depends on the size of the system, whereas for this model with disordered external fields, the excited-state fidelity exhibits a pronounced drop at the transition and then increases to approximately approach 1 in the localized phase. These findings demonstrate that disordered nearest-neighbour couplings, disordered next-nearest-neighbour couplings and disordered external fields have different effects on the MBL transition. In particular, we also investigate the MBL transition of such a quantum Ising chain with both disordered nearest-neighbour couplings and disordered next-nearest-neighbour couplings to investigate how these two types of disordered couplings drive the occurrence of the MBL transition. The results show that under these dynamics, E[*F*] decays substantially within a certain range of the (*J*, *K*) parameter space and then gradually tends towards stability. We hope that the present work can provide a meaningful tool for gaining a better understanding of the MBL transition and ergodicity breaking in quantum systems, and we will research this interesting phenomenon further in our future work.
